# Anti-Bacterial Activity of Phenolic Compounds against *Streptococcus pyogenes*

**DOI:** 10.3390/medicines4020025

**Published:** 2017-05-01

**Authors:** Sabrina Macé, Lisbeth Truelstrup Hansen, H.P. Vasantha Rupasinghe

**Affiliations:** 1 Department of Plant, Food, and Environmental Sciences, Faculty of Agriculture, Dalhousie University, Truro, NS B2N 5E3, Canada; sabmace@gmail.com; 2 Department of Process Engineering and Applied Science, Faculty of Engineering, Dalhousie University, Halifax, NS B3H 4R2, Canada; litr@food.dtu.dk; 3 Department of Pathology, Faculty of Medicine, Dalhousie University, Halifax, NS B3H 4H7, Canada

**Keywords:** pharyngitis, strep throat, biofilm, naphthoquinone, infection, disease, polyphenols

## Abstract

**Background:** Worldwide, *Streptococcus pyogenes* is the leading cause of bacterial pharyngitis. To reduce the use of antibiotics, antimicrobial phytochemical-containing remedies, which have long been in use in traditional medicine, may provide new approaches for management of streptococcal pharyngitis. The objective of this study was to assess the inhibitory activities of 25 natural phenolic compounds against three strains of *S. pyogenes*. **Methods:** After an initial screening, the minimum inhibitory concentration (MIC) and minimum bactericidal concentration (MBC) of the nine most effective phenolic compounds were determined. The effect of four compounds with the lowest MIC and MBC on streptococcal growth and biofilm formation was also studied. **Results:** 1,2-Naphthoquinone and 5-hydroxy-1,4-naphthoquinone elicited the greatest anti-*S. pyogenes* activities with MICs ranging from 0.39 to 6.25 µg mL^−1^ and MBCs of 100 µg mL^−1^. Both naphthoquinones inhibited the biofilm formation at concentrations ranging from 12.5 to 50 µg mL^−1^. Biofilm reduction and altered bacterial cell structures were visible in scanning electron microscopy images of naphthoquinone-treated cells. **Conclusion:** In conclusion, 1,2-naphthoquinone and 5-hydroxy-1,4-naphthoquinone inhibit *S. pyogenes* and should be further investigated as candidates for the management of streptococcal pharyngitis.

## 1. Introduction

Pharyngitis or bacterial infection of the throat is a common disease, which is often caused by *Streptococcus pyogenes* [1,2]. Worldwide, *S. pyogenes* are responsible for an estimated 600 million cases of throat infections per year [3]. β-Lactams, such as penicillin, are the preferred antibiotics in the treatment of *S. pyogenes* throat infections, with macrolides being used for patients with β-lactam hypersensitivity. However, while resistance to β-lactams has so far not emerged in *S. pyogenes,* resistances were found for macrolides and some quinolones (fluoroquinolone) [3,4]. Moreover, *S. pyogenes* forms biofilm, which has been associated with antibiotic treatment failures [5]. Thus, there is an urgent need to find new potent antibacterial and anti-biofilm agents which could find use in alternative and/or complementary therapy for streptococcal pharyngitis. 

In addition to treatment with prescribed medications, throat lozenges containing chemical anesthetics and antiseptics are being used by patients in the relief of the discomfort associated with the infection. The antimicrobial quaternary ammonium compound, dequalinium chloride, is similarly used in the treatment of common infections of the mouth and throat and incorporated in candy-based lozenge formulations [6]. The antiseptic and local anesthetic hexylresorcinol has also been included as an active ingredient in throat lozenges [7]. 

Natural products derived from plants have been used in traditional medicine since the ancient times and are, now, being widely studied for incorporation into mainstream products. Plant-derived compounds of interest are mostly secondary metabolites, which possess antimicrobial properties against microbial pathogens and spoilers [8]. Recently, a diverse range of phytochemical antibacterial agents has been reported to suppress the growth of *S. pyogenes*, including polyphenols (i.e., flavonoids) and terpenes [9,10,11,12]. A recent review describes the mechanisms of inhibition of different *Streptococcus* species, including *S. pyogenes*, by phytochemicals through the prevention of the bacterial adherence of the pharynx, inhibition of glycolytic enzyme and pH drop, reduction of biofilm, and alteration of cell surface hydrophobicity [2].

The objective of the present study was to identify, among 25 different commercially available plant-derived phenolic compounds, the ones with anti-*S. pyogenes* activities, which could be incorporated in dehydrated honey throat lozenges and similar products.

## 2. Material and Methods

### 2.1. Bacterial Strains and Growth Conditions

The experiments used the following human *S. pyogenes* strains: *S. pyogenes* ATCC ^®^ 19615 ™, *S. pyogenes* ATCC ^®^ 49399 ™ and a clinical isolate obtained from a patient with pharyngitis (Bacteriology, Queen Elizabeth II Hospital, Halifax, NS, Canada). The strains were routinely cultured in Brain Heart Infusion (BHI) (Oxoid, Thermo Fisher Scientific, Waltham, MA, USA) media at 37 °C under aerobic conditions. 

### 2.2. Compounds

Twenty-five phenolic compounds belonging to 12 different classes: three simple phenols (eugenol, thymol, and pyrocatechol), three isoflavones (daidzin, daidzein, and genistein), one chalcone (phloretin), one chalcone glycoside (phlorizin), two flavan-3-ols (epicatechin and epigallocatechin gallate, EGCG), two flavanones (naringenin and hesperidin), two flavones (flavone and naringin), two flavonols (myricetin and quercetin hydrate), one flavonol glucoside (quercetin-3-*O*-glucoside), one hydroxybenzoates (gallic acid), three hydroxycinnamates (*tran*-ferulic acid, cinnamaldehyde, and warfarin), two naphthoquinones (1,2-naphthoquinone and 5-hydro-1,4-naphthoquinone), one stilbene (resveratrol), one tannin (tannic acid), and two chemical compounds (used as positive controls), one quaternary ammonium compound (dequalinium chloride), and one substituted phenol (4-hexylresorcinol) were evaluated for their antibacterial properties. The compounds were purchased from Sigma-Aldrich Company (Saint-Louis, MO, USA). Before each experiment, the stock solutions were freshly made to a concentration of 10 mg mL^−1^ in 100% DMSO.

### 2.3. Preliminary Screening Using the Micro-Dilution Broth Antibacterial Assay

The inhibitory effect of the phenolic compounds (100 µg mL^−1^) was initially assessed on *S. pyogenes* ATCC 19615 and ATCC 49399, using the broth micro-dilution assay as described by Clinical and Laboratory Standards Institute [13]. Briefly, in a 96-well micro-plate, 2 µL of the 100 mg mL^−1^ stock solution was added to 98 µL of BHI broth in order to reach a final test compound concentration of 100 µg mL^−1^. Finally, for each strain, 100 µL of standardized inoculum was added, resulting in an initial bacterial concentration of 5 log_10_ (CFU mL^−1^). Bacterial inocula in BHI broth (2 µL water) and BHI broth +1% DMSO were also run as a control. The growth of the bacteria was assessed after incubation for 24 h at 37 °C by reading the A_600_ nm using a plate reader (Epoch ^TM^, Biotek, Winooski, VT, USA). Each test was done in triplicate. In order to determine the most active compounds, a decrease in the A_600_ nm of 0.1 or more in the presence of the tested compounds in comparison to the control (bacteria inocula in BHI +1% DMSO) was considered to be indicative of an inhibition of the growth.

### 2.4. Minimum Inhibitory Concentration (MIC) and Minimum Bactericidal Concentration (MBC)

MIC broth micro-dilution assay was performed as described in the previous section, except 2-fold dilutions were used to yield final test compound concentrations ranging from 0.19 to 100 µg mL^−1^, on the three *S. pyogenes* strains. The MIC was determined as the first concentration where the decrease of A_600_ nm was significant (*p* < 0.05) in comparison with the control. To determine MBC, 30 µL from each well, where no growth was detected—i.e., compound concentration ≥MIC—was spread on BHI agar plates and incubated for 24–48 h at 37 °C. The lowest compound concentration resulting in no growth on the agar plate represented the MBC. Each sample was tested in triplicate and each experiment was repeated three times.

### 2.5. Time-Kill Assay 

The effect of concentrations ranging from 1 to 16 times MIC of the four most active compounds on the growth of *S. pyogenes* ATCC 19615 was quantified after 0, 2, 4, 6, 8, 10, and 24 h at 37 °C. At each time point, an aliquot (100 μL) was pipetted, serially diluted, and plated on BHI agar followed by enumeration and calculation of the mean log_10_ CFU mL^−1^ of the duplicate samples. Each time-kill experiment was repeated in three biologically independent assays (*n* = 6).

### 2.6. Inhibition of Biofilm Formation

The ability of *S. pyogenes* to form biofilm in the presence of 1,2-naphthoquinone, 5-hydroxy-1-naphthoquinone, dequalinium chloride, or 4-hexylresorcinol (0.19–100 µg mL^−1^) was assessed by measuring the metabolic activity of sessile biofilm cells using a 3-(4,5-dimethylthiazol-2-yl)-2,5-diphenyltetrazolium bromide (MTT) dye assay. After an exposure period of three days at 37 °C, the planktonic bacterial cells were eliminated by carefully removing the BHI medium from the wells. Adapted MTT assay was performed as described in Vybrant^®^ MTT Cell Proliferation Assay Kit Protocol (Thermo Fisher Scientific, Waltham, MA, USA). After incubation for 10 min at 37 °C, the A_540_ nm was measured and used to calculate the minimum biofilm inhibitory concentration (MBIC), defined as the first concentration where the decrease in A_540_ nm was significant (*p* < 0.05) in comparison with the control.

### 2.7. Scanning Electron Microscopy (SEM)

*S. pyogenes* ATCC 19615 biofilms formed (72 h at 37 °C) in the presence of MBIC concentrations of 1,2 naphthoquinone, 5-hydroxy 1,4 naphthoquinone, dequalinium chloride, 4-hexylresorcinol were prepared in microplates. The *S. pyogenes* biofilms were also grown in BHI broth (water) and BHI broth +1% DMSO as controls. After incubation, a sample fixation method for SEM [14] adapted for microplate was performed. After discarding the last solution, the plate was air dried for 2 h in a fume-hood. The bottom of the wells (location of the biofilms) were then removed with a heated blade and mounted on aluminum mounts using the carbon adhesive and finally sputter-coated with gold and examined using a Hitachi S-4700 SEM (Tokyo, Japan).

### 2.8. Statistical Analysis

During the determination of the MIC and MBIC, statistically significant differences (Student *t*-test (paired, two tailed), *p* < 0.05) between test compound concentrations and controls were determined using the Student *t*-test on the A_600_ nm or A_540_ nm data, respectively.

## 3. Results

The results of the screening test against two *S. pyogenes* strains, ATCC 19615 and ATCC 49399 showed that nine compounds exhibited antibacterial activity against at least one strain at the concentration of 100 µg mL^−1^. Six of them, phlorizin, naringenin, myrcetin 1,2-naphthoquinone, 5-hydroxy-1,4-naphthoquinone, and resveratrol inhibited both strains like the two positive controls. Flavone and thymol affected the growth of strain ATCC 19615 while cinnamaldehyde inhibited only ATCC 49399. 

The MIC of those nine phytochemicals (Table 1) varied from 0.39 µg mL^−1^ to >100 µg mL^−1^ and the MBC from 100 µg mL^−1^ to >100 µg mL^−1^ with some sensitivity/resistance variations among strains. 1,2-Naphthoquinone and 5-hydroxy-1,4-naphthoquinone exhibited the best inhibitory effect with similar MIC values relative to the positive controls and were selected for further analysis of their effect on bacterial growth and biofilm formation.

**Figure 1:**
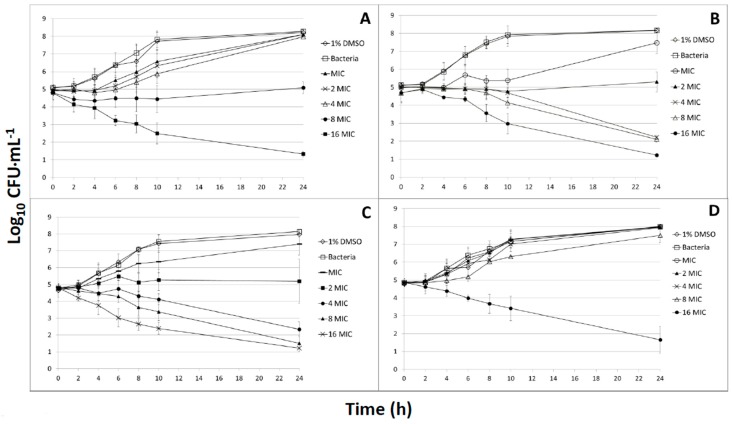
The effect of several concentrations of the four compounds on the growth of *S. pyogenes* ATCC 19615 at 37 °C: (**A**) 1,2-naphthoquinone (MIC: 3.125 µg mL^−1^); (**B**) 5-hydroxy-1,4-naphthoquinone (MIC: 1.56 µg mL^−1^); (**C**) dequalinium chloride (MIC: 0.39 µg mL^−1^); and (**D**) 4-hexylresorcinol (MIC: 1.56 µg mL^−1^). Arrows indicate that plate count values were below the detection threshold of 1.22 log_10_ CFU mL^−1^.

The four compounds that higher concentrations increased the lag phase and the bacterial log_10_-reduction (Figure 1). The presence of low concentrations (<12.5 µg mL^−1^) of 1,2-naphthoquinone delayed growth during the first 10 h but after 24 h the bacterial concentration reached around 8 log_10_ CFU mL^−1^ (Figure 1A). A concentration of 25 µg mL^−1^ displayed a bacteriostatic effect for the first 10 h, after which growth resumed to reach 5 log_10_ CFU mL^−1^ after 24 h. At 50 µg mL^−1^, the growth was inhibited and a bacterial log_10_-reduction of more than 3.5 log_10_ CFU mL^−1^ observed after 24 h.

Administration of 1.56 µg mL^−1^ 5-hydroxy-1,4-naphthoquinone caused populations to remain under 6 log_10_ CFU mL^−1^ for 10 h; however, after 24 h more than 7 log_10_ CFU mL^−1^ was reached (Figure 1B). A bacteriostatic effect was similarly observed with 3.125 µg mL^−1^ 5-hydroxy-1,4-naphthoquinone where bacterial counts were under 6 log_10_ CFU mL^−1^ after 24 h. Using concentrations of 6.25 and 12.5 µg mL^−1^, numbers stayed around 4–5 log_10_ CFU mL^−1^ for 10 h and then dropped about 2 log_10_ CFU mL^−1^. With 25 µg mL^−1^, the bacterial concentrations underwent a 2 log_10_-reduction after 10 h and reached the limit of detection (>1.22 log_10_ CFU mL^−1^) after 24 h.

For the dequalinium chloride (Figure 1C), the same trend of inhibition was observed where the lowest test concentrations of 0.39 µg mL^−1^ permitted growth and a twice-higher concentration of 0.78 µg mL^−1^ displayed a bacteriostatic effect. After 24 h, the concentrations of 1.56 and 3.125 µg mL^−1^ induced more than 2.5 log_10_ CFU mL^−1^ and 6.25 µg mL^−1^ caused a >4 log_10_-reduction (below the detection limit). 

The concentrations of 4-hexylresorcinol ranging from 1.56 to 6.25 µg mL^−1^ caused a growth delay and a small log_10_-reduction (under 1 log_10_ CFU mL^−1^) after 24 h. However, the concentration of 25 µg mL^−1^ induced a log_10_-reduction of more than 3 log_10_ CFU mL^−1^ after 24 h (Figure 1D). 

The two naphthoquinones as well as the substituted phenol, 4-hexylresorcinol, inhibited biofilm formation with MBICs of between 25–50 µg mL^−1^ for 1,2-naphthoquinone, 12.5–50 µg mL^−1^ for 5-hydroxy-1,4-naphthoquinone, and from 12.5 to 25 µg mL^−1^ for 4-hexylresorcinol (Table 1). Dequalinium chloride displayed the lowest MBIC ranging from 0.39–0.78 µg mL^−1^. The sensitivity to the inhibition of biofilm formation varied among the strains and depended on the compounds. ATCC 19615 was more sensitive to 1,2-naphthoquinone but more resistant to 4-hexylresorcinol than the two other strains (Table 1).

**Figure 2:**
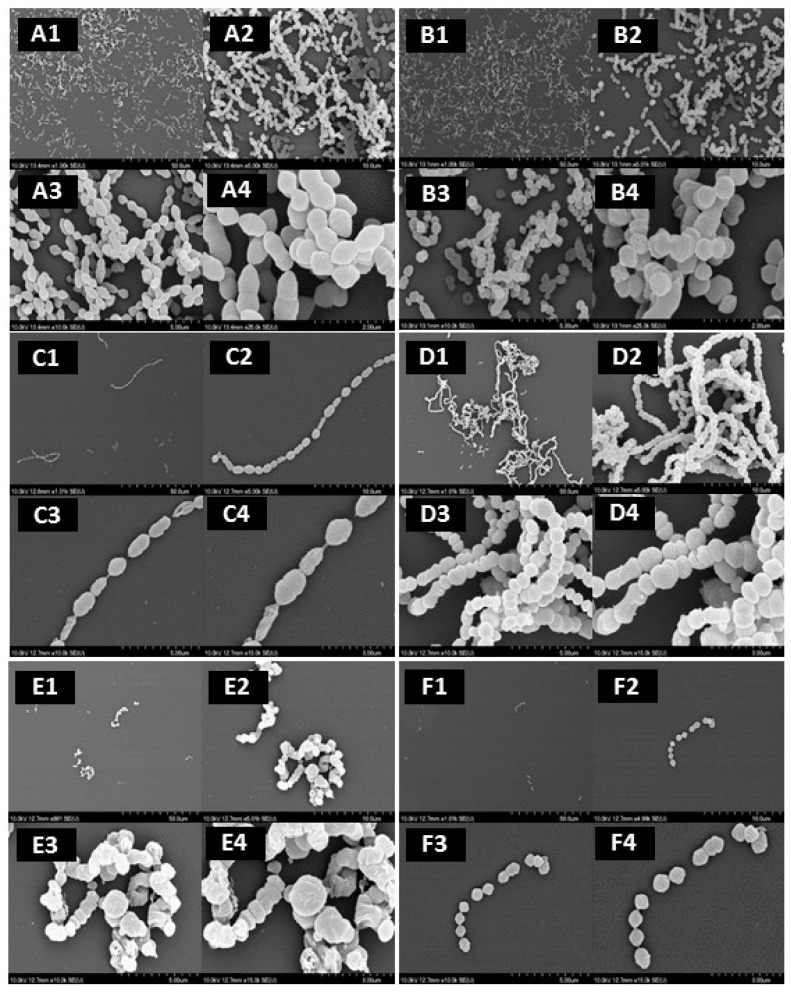
Scanning electron microscopy images of the effect of MBIC concentrations of the compounds on the biofilm formation by strain *S. pyogenes* ATCC 19615 at 37 °C: *S. pyogenes* in BHI (**A**) and with 1% DMSO (**B**); *S. pyogenes* with 25 µg mL^−1^ of 1,2 naphthoquinone (**C**); 12.5 µg mL^−1^ of 5-hydroxy-1,4 naphthoquinone (**D**); 0.78 µg mL^−1^ of dequalinium chloride (**E**) and 25 µg mL^−1^ of 4-hexylresorcinol (**F**). Magnification value: I, ×1000; II, ×5000; III, ×1000; IV, ×15,000; V, ×25,000.

SEM images showed the visual reduction of *S. pyogenes* ATCC 19615 biofilm in the presence of the MBIC of the four compounds by strongly reducing the concentration of the attached cells and modifying their shape (Figure 2). The presence of 1% DMSO in BHI (Figure 2B) reduced the chain length of the cocci and biofilm density, and also appeared to shrink the cells slightly in comparison to BHI only treatment (Figure 2A). Figure 2C shows that 25 µg mL^−1^ of 1,2-naphthoquinone reduced strongly the concentration of the attached cells, leaving only a few long and thin chains comprised of shrunken, irregular cells. In the presence of 12.5 µg mL^−1^ of 5-hydroxy-1,4-naphthoquinone (Figure 2D), smaller cells aggregated in clusters of chains. Figure 2E presents the effect of dequalinium chloride at 0.78 µg mL^−1^, which induced the formation of very small groups of bacterial cells and completely altered their shape. The MBIC of 4-hexylresorcinol (Figure 2F) affected the chain formation of the bacteria and only a few small chains containing 2–4 cells were observed. 

## 4. Discussion

According to the literature, the MICs of phenolic compounds against pharyngitis pathogens range from 1.52 to 100 µg mL^−1^ [9,10,11,12]. Among all the phenolic compounds tested in this study, nine compounds showed an anti-Streptococcal activity at a concentration lower than 100 µg mL^−1^. Gyawali and Ibrahim. (2014) [8] stated that the hydroxyl (-OH) group in phenolic compounds may cause bacterial inhibition and described the importance of double bonds (number and position) in relation to antimicrobial effectiveness. Inferences could be made to the present study as, among the nine active compounds, the most anti-*S. pyogenes* naphthoquinones possess two carbonyl groups in an aromatic ring as part of their quinone structure. This hypothesis is supported by a recent review which associated the oxydo-reduction activity of the quinone structure in 2-hydroxy-1,4-naphthoquinone with the generation of reactive oxygen species (ROS) and damage of macromolecules such as DNA, proteins, and lipids [15]. Ndi et al. (2007) [11] isolated another naphthoquinone from *Eremophila serrula,* which also exhibited antagonism against *S. pyogenes* strain ATCC 10389 with low MIC (7.8 µg mL^−1^) and MBC (15.6 µg mL^−1^). 

Clinically, most antibacterials are described as potentially being both bactericidal and bacteriostatic. This was also observed in this study as the tested compounds. Even if a bactericidal action is preferred in the context of treatment, achieving a bacteriostatic effect may advantageously inhibit exotoxin production in *Staphylococci* and *Streptococci* thereby avoiding induction of the toxic shock syndrome [16]. In comparison with the kinetics of the tested compounds, rhodomyrtone—the potent bioactive compound from *Rhodomyrtus tomentosa*—appears to be slightly more effective: it exerted a faster bactericidal activity on *S. pyogenes* (within 5 and 6 h) at lower concentrations (6.5–12.5 µg mL^−1^) [17]. However, rhodomyrtone (Sigma-Aldrich Co., Saint-Louis, MO, USA) is about 2000–4500 times more expensive than the naphthoquinones tested. 

To the best of our knowledge, the anti-biofilm potential of naphthoquinones against *S. pyogenes* has not been explored. The inhibition of biofilm formation is an interesting way to prevent the formation of well-organized attached bacterial biofilms and, thus, pharyngitis. We observed that the naphthoquinones elicited significant anti-biofilm activity against *S. pyogenes*, with metabolic MBICs in the same range as 4-hexylresorcinol and this was confirmed by SEM, where *S. pyogenes* ATCC 19,615 cells appeared less aggregated, showed morphological changes, and reduced biofilm formation. These observations are in accordance with the potential of phenolic compounds to damage the membrane structure as well as anti-*S. pyogenes* properties of phytochemicals such as anti-adhesion, anti-biofilm, and quorum-sensing inhibition [2,8]. The generation of ROS during the bio-reduction of naphthoquinones [15] might be linked to the loss of cell wall integrity observed. In addition, the visible effect of 1% DMSO on the bacteria, which do not appear on the growth (Figure 1), could also slightly enhance the antibacterial effect of the phytochemicals.

Considering the potential clinical application of our study, additional experiments could be conducted on combination of natural antibacterial agents and currently used antibiotics to enhance the present management practices against *S. pyogenes.* If the combination demonstrates a synergic and/or a complementary effect (e.g., anti-inflammatory and antitussive), it could become a combined treatment for patient’s pain relief. Moreover, it is also possible to incorporate the efficacious natural compounds in dehydrated honey lozenges. Honey itself has a recognized effect as being systemic antitussive and antimicrobial natural source [18,19,20]. Interestingly, a recent study reported that honey with a high phenolic content presented the highest antimicrobial activity [20]. Further investigations are required to affirm the synergic effect with honey but also understand the safety of the use of naphthoquinones in throat lozenges before making any recommendations for their use for managing streptococcal pharyngitis.

## Figures and Tables

**Table 1:** Minimum inhibitory concentration (MIC, µg mL^−1^), minimum bactericidal concentration (MBC, µg mL^−1^) of 11 compounds and minimum biofilm inhibitory concentration (MBIC, µg mL^−1^) of the four most active compounds against three different strains of *S. pyogenes.*

Streptococcus Pyogenes Strains Compounds	Chemical Structure	ATCC 19615			ATCC 49399			A Clinical Isolate		
		MIC	MBC	MBIC	MIC	MBC	MBIC	MIC	MBC	MBIC
Phlorizin	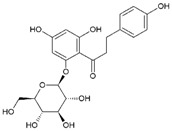	100	>100 ^a^	nd	100	>100	nd	100	>100	nd
Naringenin	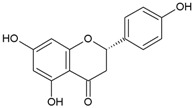	50	>100	nd	50	>100	nd	50	>100	nd
Flavone	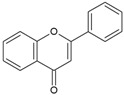	50	>100	nd	>100	>100	nd	25	>100	nd
Myricetin	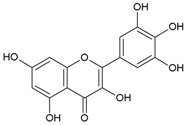	50	>100	nd	100	>100	nd	100	>100	nd
Thymol	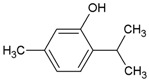	25	>100	nd	>100	>100	nd	≥100	>100	nd
1,2-Naphthoquinone	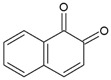	3.125	100	25	6.25	100	50	6.25	100	50
5-Hydroxy-1,4-naphthoquinone	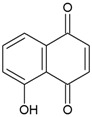	1.56	100	12.5	1.56	>100	50	0.39	100	12.5
Resveratrol	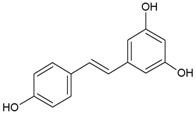	100	>100	nd	50	>100	nd	>100	>100	nd
Cinnamaldehyde	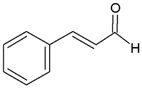	>100	>100	nd	50	>100	nd	25	>100	nd
Dequalinium chloride	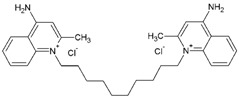	0.39	12.5	0.78	0.78	25	0.78	0.78	12.5	0.39
4-Hexylresorcinol	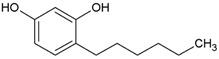	1.56	50	25	6.25	50	12.5	12.5	50	12.5

^a^ MBC was higher than the maximum concentration of 100 µg mL^−1^ used in this study; nd: not-determined.
